# RETRACTION: Monodispersed Copper(I)‐Based Nano Metal–Organic Framework as a Biodegradable Drug Carrier With Enhanced Photodynamic Therapy Efficacy

**DOI:** 10.1002/advs.75902

**Published:** 2026-05-28

**RETRACTION**: X. Cai, Z. Xie, B. Ding, S. Shao, S. Liang, M. Pang, and J. Lin, “Monodispersed Copper(I)‐Based Nano Metal–Organic Framework as a Biodegradable Drug Carrier with Enhanced Photodynamic Therapy Efficacy,” *Advanced Science* 6, no. 15 (2019): 1900848, https://doi.org/10.1002/advs.201900848.

The above article, published online on 22 May 2019 in Wiley Online Library (wileyonlinelibrary.com), and has been retracted by agreement between the authors; the journal Editor‐in‐Chief, Kirsten Severing; and Wiley‐VCH GmbH. Concerns were raised by a third party on PubPeer [1] that panels in Figure 4e contained overlapping sections and that the mice shown for the Control and PBS+Light images had been duplicated in Figure 4D.

Further investigation by the publisher found that the Control and CuTz‐1‐O_2_@F127 Lung images in Supplementary Figure 15 also contained the same overlapping section and that Figure S1 contains a higher magnification image of the sample also reported in Supplementary Figure 16b, though both images represent different samples. Additionally, a manipulated version of the CuTz‐1‐O_2_@F127 Kidney image was re‐used in a later article by a different author group [Wang et al. 2020 (https://doi.org/10.1039/C9RA09860G)]. Lastly, the investigation found that the article did not provide sufficient information about ethics committee approval for the animal experiments reported in the article.

The authors responded to an inquiry by the publisher. While the authors shared data and images corresponding to the experiments reported in this article, the authors also confirmed that the original ethics approval documentation is no longer available.

The editors have concluded that the lack of information regarding ethics committee approval for animal experiments violates the journal's and the publisher's ethics guidelines. Therefore, all parties agree that the article must be retracted. The authors were informed about the retraction.
